# A Novel and Simple Score to Predict Embolic or Atherosclerotic Middle Cerebral Artery Occlusion Before Mechanical Thrombectomy: AHOC Score

**DOI:** 10.1002/cns.70729

**Published:** 2025-12-31

**Authors:** Hudie Zhang, Yingwen Su, Zubing Xu, Yunqing Chen, Rongwei Yang, Weiming Gan, Zhaojun Huang, Laisheng Cai, Chenying Zeng, Qin Huang, Jinchong Zhang, Keji Zou, Jingwei Huang, Pu Fang, Xiaobing Li, Yuhua Fan, Daojun Hong, Jing Lin

**Affiliations:** ^1^ Department of Neurology The First Affiliated Hospital, Jiangxi Medical College, Nanchang University Nanchang Jiangxi China; ^2^ Institute of Neurology, Jiangxi Academy of Clinical Medical Science, The First Affiliated Hospital Jiangxi Medical College, Nanchang University Nanchang Jiangxi China; ^3^ Rare Disease Center, The First Affiliated Hospital, Jiangxi Medical College Nanchang University Nanchang Jiangxi China; ^4^ Key Laboratory of Rare Neurological Diseases of Jiangxi Provincial Health Commission, Jiangxi Medical College Nanchang University Nanchang Jiangxi China; ^5^ Department of Neurology National Key Clinical Department and Key Discipline of Neurology, The First Affiliated Hospital of Sun Yat‐Sen University Guangzhou Guangdong China

**Keywords:** embolism, intracranial arteriosclerosis, mechanical thrombectomy, middle cerebral occlusion, scoring system

## Abstract

**Objective:**

The mechanical thrombectomy (MT) strategy obviously differs for acute middle cerebral artery occlusion (MCAO) stroke caused by embolism or atherosclerosis. Our study aimed to develop and validate a simple and universally applicable score for predicting etiology [embolism or intracranial arteriosclerosis (ICAS)] before MT in patients with acute MCAO stroke.

**Methods:**

Between November 2019 and September 2022, we retrospectively enrolled eligible patients in our hospital as the training cohort. Additionally, consecutive patients between July 2023 and April 2024 were recruited as the validation cohort. Multivariate logistic regression analysis was used to identify the independent factors associated with etiology in the training group. Each factor was then point assigned based on β‐coefficient, and a risk scoring system was developed. The scoring system was validated through the validation cohort. The *C*‐statistic, Brier score, and Hosmer‐Lemeshow test were used to assess model discrimination and calibration.

**Results:**

The training group and validation group finally included 277 patients (154 embolism‐MCAO and 123 ICAS‐MCAO) and 101 patients (59 embolism‐MCAO and 42 ICAS‐MCAO), respectively. A scoring system (AHOC score) covering four variables (atrial fibrillation, hyperdense middle cerebral artery sign, stenosis/occlusion in other arteries, and collateral status) was derived to help identify embolism‐MCAO or ICAS‐MCAO. The AHOC score showed good discrimination and calibration in the training cohort (*C*‐statistic, 0.932 [0.902–0.963]; Brier score, 0.092 [0.070–0.115]; *p* value of the Hosmer‐Lemeshow test, 0.604) and in the validation cohort (*C*‐statistic, 0.933 [0.888–0.978]; Brier score, 0.102 [0.067–0.140]; *p* value of the Hosmer‐Lemeshow test, 0.846). According to the AHOC score, patients with a score of 4–8 were identified as high‐risk for the embolism‐MCAO category. Conversely, a patient with a score of 0–3 was considered high‐risk for the ICAS‐MCAO category.

**Conclusions:**

Our scoring system (AHOC score), consisting of atrial fibrillation, hyperdense middle cerebral artery sign, stenosis/occlusion in other arteries and collateral status, is a valid and applicable model for predicting the etiology in patients with acute MCAO before MT.

## Graphical abstract

This study developed and validated the novel AHOC score, a simple pre‐thrombectomy tool for predicting stroke etiology (embolism vs. intracranial atherosclerosis) in acute middle cerebral artery occlusion. This score integrates four key clinical/imaging variables—atrial fibrillation, hyperdense artery sign, stenosis/occlusion in other arteries, and collateral status—and demonstrates excellent predictive accuracy. It aids in tailoring mechanical thrombectomy strategy by identifying the underlying cause preoperatively.

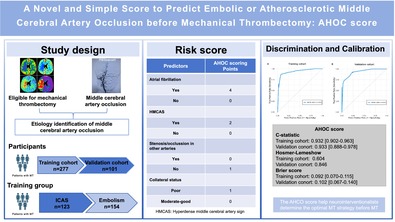

## 1 Introduction

The global burden of disease data shows that stroke is the leading cause of death and disability worldwide [1]. Several randomized controlled trials have consistently demonstrated the benefit of mechanical thrombectomy (MT) within 24 h of stroke onset in patients with large vessel occlusion (LVO) [2, 3, 4]. Therefore, MT has become the current standard treatment for acute ischemic stroke in patients with LVO. A multicenter prospective registry study involving 111 stroke centers in China showed that embolism‐related LVO (embolism‐LVO) and intracranial arteriosclerosis‐related LVO (ICAS‐LVO) accounted for more than 85% of all LVO cases [5].

Currently, stent retrievers and direct aspiration are the two main approaches of MT in patients with LVO, and different etiologies may determine different surgical strategies [6]. In ICAS‐LVO, up to half of patients experience reocclusion, and the recanalization rates tend to be lower than those of embolism‐LVO, often requiring further salvage angioplasty [7, 8]. Previous studies showed that stent retrievers achieved a higher rate of successful reperfusion compared to direct aspiration in ICAS‐LVO patients [9, 10]. Because of these features, stent retrievers are the first‐line approach in the treatment of ICAS‐LVO. In contrast, the efficacy of direct aspiration for recanalization is better in patients with embolism‐LVO than in those with ICAS‐LVO [9, 11]. Hence, identifying the etiology of LVO before MT can help clinicians prepare the surgical plan.

Previous studies have reported multiple factors that can predict the etiology of LVO before MT, such as hyperdense sign, leptomeningeal collateral status, atrial fibrillation, and so on [12, 13, 14]. However, some indicators may be applicable to specific large vessel occlusions, such as the hyperdense sign that is most evident in the middle cerebral artery and may be false negative if used for etiological judgment of other large vessel occlusions [13]. On the other hand, collateral evaluation is less suitable for large vessel occlusions of the posterior circulation [14]. Therefore, predictors for the etiology of LVO vary between the anterior and posterior circulations or between different vessels.

Although middle cerebral artery occlusion (MCAO) is the most common type of large vessel occlusion stroke, there is no specific predictive model for MCAO etiology. Therefore, the current study aimed to construct and validate a simple and universally applicable score for etiology before MT in patients with MCAO.

## 2 Methods

### 2.1 Patients' Selection

In the training group, we retrospectively collected patients with acute large vessel occlusion stroke who were admitted to the First Affiliated Hospital of Nanchang University between November 2019 and September 2022. Additionally, consecutive acute ischemic stroke patients with large vessel occlusion at the First Affiliated Hospital of Nanchang University between July 2023 and April 2024 were recruited as a validation cohort. The sample size of the validation cohort was determined by the number of consecutive eligible patients presenting to our center during the predefined prospective recruitment period (July 2023 to April 2024), which reflects the real‐world clinical workflow and logistical constraints of a single‐center study. All procedures were approved by the Ethics Committee of the First Affiliated Hospital of Nanchang University.

Patients were eligible for the current study if they met the following criteria: (1) age 18 years or older; (2) presented within 24 h of symptom onset; (3) acute ischemic stroke with single middle cerebral artery (MCA) occlusion (M1, M2, or M3 segment); (4) received mechanical thrombectomy with or without intravenous thrombolysis; (5) underwent baseline non‐contrast computed tomography (CT) and CT perfusion (CTP). Patients were excluded if they met the following criteria: (1) the cause of middle cerebral artery occlusion was vasculitis, dissection, moyamoya disease, or other identified etiologies; (2) unidentified pathogenesis of occluded middle cerebral artery; (3) tandem lesions associated with the carotid artery; (4) missing digital subtraction angiography (DSA) imaging information; (5) incomplete baseline or follow‐up data.

### 2.2 Data Collection

Demographic characteristics and clinical variables were recorded, including age, sex, history of hypertension, history of diabetes, history of stroke, presence or absence of atrial fibrillation, admission systolic blood pressure (SBP) and diastolic blood pressure (DBP), baseline National Institute of Health Stroke Scale (NIHSS) scores, time from onset to admission, time from onset to completion of CTP, puncture to reperfusion time (PRT), and door to puncture time (DPT). Laboratory tests within 24 h after mechanical thrombectomy were collected, including platelet, lymphocyte count, monocyte count, neutrophil count, uric acid, fasting glucose, fibrinogen, and D‐dimer. Furthermore, the neutrophil to lymphocyte ratio (NLR), lymphocyte to monocyte ratio (LMR), and platelet to lymphocyte ratio (PLR) were calculated. Moreover, a 3‐month follow‐up was performed through telephone and modified Rankin Scales at 3 months were recorded.

### 2.3 Imaging Protocol and Analysis

All patients eligible for the present study underwent a standardized imaging evaluation before mechanical thrombectomy. CTP images were acquired on the Siemens Somatom Force (Erlangen, Germany), and all scans were done with 40 mL of nonionic iodinated contrast (Iomeron, iomeprol, 400 mg iodine/mL; Patheon Italia S.P.A., Ferentino, Frosinone, Lazio, Italy). All CTP datasets were post‐analyzed using the software package (Syngo.via CT Neuro Perfusion VB40). The thresholds for core and hypoperfusion tissue were defined as relative cerebral blood flow (rCBF) < 30% and TMax > 6 s, respectively. Finally, the core volume, penumbra volume, and mismatch ratio were calculated and collected. More importantly, we measured a relatively novel marker, the early infarct growth rate (EIGR), to reflect the rate of early infarct core growth. EIGR was calculated using the following formulas: EIGR = core volume (mL)/time from onset to completion of CTP (hours) [15]. Alberta stroke program early computed tomography score (ASPECTS) and hyperdense middle cerebral artery sign (HMCAS) were evaluated through non‐contrast CT. HMCAS was defined as a density of 53–69 Hounsfield units in the middle artery [16].

To assess the presence of moderate to severe stenosis/occlusion of other cervicocranial arteries and collateral status before MT, sequential DSA images were analyzed. The presence of a stenosis of 50% or more or occlusion in the other cervicocranial arteries except the responsible middle cerebral artery was defined as outcome variable with stenosis/occlusion in other arteries. Collateral status was graded as poor, moderate, and good according to the American Society of Intervention and Therapeutic Neuroradiology/Society of Interventional Radiology (ASITN/SIR) collateral scale [17, 18]. All radiological assessments were performed by two trained neurologists who were blind to patients' information.

### 2.4 Definition

If the culprit occluded MCA achieved successful reperfusion (residual stenosis < 50%) after primary thrombectomy without atherosclerotic evidence or had a definite source of embolism, the etiology was defined as middle cerebral occlusion due to embolism (Embolism‐MCAO) [19]. However, patients with obvious stenosis (> 50% stenosis of the culprit MCA) after primary thrombectomy or re‐occlusion tendency after successful reperfusion were considered as middle cerebral occlusion due to intracranial atherosclerosis (ICAS‐MCAO) [19]. In addition, symptomatic hemorrhagic transformation (HT) was defined as hemorrhage seen on CT with an increase in the NIHSS score (≥ 4 points) [20].

## 3 Statistical Analysis

Continuous variables are presented as the mean ± standard deviation, or median [interquartile range (IQR)] dependent on the distribution. The student's *t* test or Mann–Whitney *U* test was used to compare continuous variables between groups. Categorical variables were presented as numbers and frequency *N* (percentages) and compared using the Chi‐square test or Fisher exact test.

Univariate and multivariate logistic regression analyses were used to determine the independent predictors of etiology classification in the training cohort. Continuous variables with significant differences in univariate analysis were divided into dichotomous variables based on cutoff values using receiver operating characteristic (ROC) curve analysis. The predictive abilities of infarct core volume, mismatch volume, mismatch ratio, and EIGR were compared using ROC curves, and then the variable with the strongest predictive power was included in the multivariate logistic regression analysis. Subsequently, variables that were independently associated with the etiology in the multivariate logistic regression model and exhibited no substantial multicollinearity (variance inflation factors, VIF < 5) were included in the final scoring system. The risk score system was subsequently generated and a score was assigned for each risk factor based on the Framingham Study [21]. The optimal cutoff value for the final score was determined through ROC curve analysis and the maximization of Youden's Index. The score was dichotomized into Embolism‐MCAO and ICAS‐MCAO categories based on optimal cutoff value. Patients scoring optimal cutoff points or higher were classified as being at high risk for Embolism‐MCAO. Conversely, patients scoring less than optimal cutoff points were classified as being at high risk for ICAS‐MCAO.

Calibration was assessed by the Hosmer‐Lemeshow test and the Brier score to determine goodness of fit. Discrimination was measured by the *C* statistic to predict accuracy. The diagnostic performance of this binary classification was assessed by calculating the following metrics: positive predictive value (PPV) and negative predictive value (NPV) with their respective 95% confidence intervals (CIs), sensitivity, and specificity.

All variables with a *p*‐value < 0.05 were considered statistically significant. All the statistical analyses were performed using SPSS (version 26.0) and R software package (version 4.2.1).

## 4 Results

### 4.1 Patients' Characteristics

**FIGURE 1:**
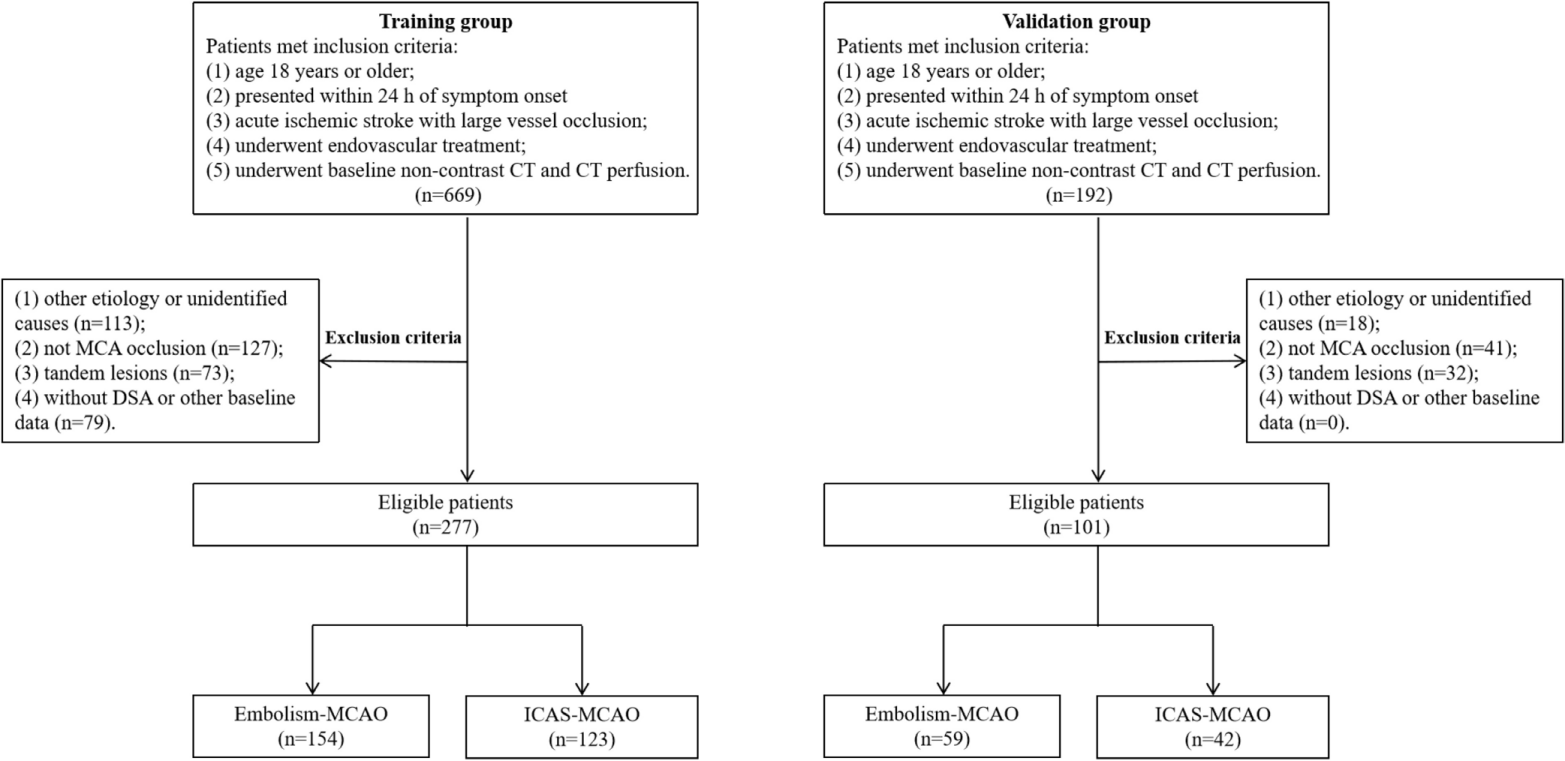
Selection of study participants in training and validation groups. CT, computed tomography; DSA, digital subtraction angiography; Embolism‐MCAO, middle cerebral artery occlusion due to embolism; ICAS‐MCAO, middle cerebral occlusion due to intracranial atherosclerosis; MCA, middle cerebral artery.

As shown in Figure 1, there were 669 patients with acute large vessel occlusion stroke retrospectively recruited between November 2019 and September 2022. After an exclusion of 113 patients with unidentified or other causes, 127 patients with non‐MCA occlusion, 73 patients who had tandem lesions, and 79 patients who lacked DSA or other baseline data. Finally, a total of 277 patients (154 embolic etiology and 123 atherosclerotic etiology) were included in the training group. The median age of the total patients in training group was 68.0 (range 58.0–76.0) years, and 59.6% were men. Additionally, between July 2023 and April 2024, 192 patients who met the inclusion criteria were screened in the validation group. Of those, 91 patients were excluded, 18 because of unidentified or other causes, 41 because of non‐MCA occlusion, and 32 because of tandem lesions. Thus 101 patients enrolled in the validation group [median age 66.0 years, 51 (50.5%) men], including 59 patients identified as embolism‐MCAO, and 42 patients considered as ICAS‐MCAO.

The clinical characteristics of the patients in the training and validation cohorts were shown in Table 1. Except for fibrinogen, no significant differences in demographic characteristics, medical history, clinical information, imaging data, laboratory tests, outcome, and stroke etiology were found between patients of the two groups.

**TABLE 1:** Comparison of baseline characteristics between training cohort and validation cohort.

Characteristics	Training cohort (*n* = 277)	Validation cohort (*n* = 101)	*p*
Demographic characteristics			
Age (year), median (IQR)	68.0 (58.0, 76.0)	66.0 (56.0, 75.0)	0.390
Male, *n* (%)	165 (59.6%)	51 (50.5%)	0.127
Medical history			
Hypertension, *n* (%)	178 (64.3%)	62 (61.4%)	0.630
Diabetes, *n* (%)	58 (20.9%)	13 (12.9%)	0.101
History of stroke, *n* (%)			
None	243 (87.7%)	88 (87.1%)	0.939
Ischemic	31 (11.2%)	12 (11.9%)	
Hemorrhagic	3 (1.1%)	1 (1.0%)	
Atrial fibrillation, *n* (%)	134 (48.4%)	39 (38.6%)	0.103
Clinical information			
Admission SBP (mmHg), median (IQR)	128.0 (115.0, 141.0)	122.0 (112.0, 136.0)	0.125
Admission DBP (mmHg), mean ± SD	73.83 ± 13.82	72.71 ± 14.74	0.496
Baseline NIHSS scores, median (IQR)	15.0 (10.0, 19.0)	14.0 (10.0, 21.0)	0.887
Onset to admission time (h), median (IQR)	4.63 (2.05, 8.17)	5.00 (2.00, 8.25)	0.806
PRT (min), median (IQR)	83.00 (53.00, 90.00)	70.00 (51.00, 100.00)	0.360
DPT (min), median (IQR)	105.00 (85.00, 140.00)	110.00 (88.00, 151.00)	0.440
Neuroimaging‐CT			
With HMCAS, *n* (%)	141 (50.9%)	47 (46.5%)	0.486
ASPECTS Classification, *n* (%)			
ASPECTS (0–5 scores)	29 (10.5%)	10 (9.9%)	1.000
ASPECTS (6–10 scores)	248 (89.5%)	91 (90.1%)	
Onset to CT time (h), median (IQR)	5.13 (2.55, 8.68)	5.50 (2.50, 8.75)	0.806
Infarct core (mL), median (IQR)	37.25 (13.84, 60.83)	27.48 (14.34, 47.63)	0.178
Penumbra median (mL), (IQR)	90.11 (48.96, 132.90)	86.54 (47.92, 123.45)	0.340
Mismatch ratio, median (IQR)	3.26 (2.25, 6.89)	3.73 (2.32, 6.46)	0.978
EIGR (mL/h), median (IQR)	8.25 (1.95, 17.85)	4.94 (2.06, 13.17)	0.330
Neuroimaging‐DSA			
Occlussion site, *n* (%)			
MCA‐M1	231 (83.4%)	86 (85.1%)	0.950
MCA‐M2	42 (15.2%)	14 (13.9%)	
MCA‐M3	4 (1.4%)	1 (1.0%)	
With stenosis/occlussion in other arteries, *n* (%)	38 (13.7%)	20 (19.8%)	0.150
Collateral circulation evaluation, *n* (%)			
Poor	79 (28.5%)	34 (33.7%)	0.529
Moderate	106 (38.3%)	33 (32.7%)	
Good	92 (33.2%)	34 (33.7%)	
Admission laboratory data			
NLR, median (IQR)	4.76 (3.32, 7.86)	4.81 (3.64, 6.72)	0.541
LMR, median (IQR)	3.64 (2.53, 4.77)	3.32 (2.47, 4.16)	0.665
PLR, median (IQR)	149.12 (109.14, 211.48)	141.79 (113.50, 196.15)	0.215
Uric acid(mmol/L), median (IQR)	357.80 (295.50, 415.40)	352.60 (314.80, 414.00)	0.814
Fasting glucose (mg/dL), median (IQR)	7.62 (6.37, 8.44)	7.09 (6.20, 8.07)	0.099
Fibrinogen (g/L), median (IQR)	2.70 (2.42, 3.48)	3.13 (2.57, 3.42)	0.019*
D‐dimer (mg/L), median (IQR)	1.02 (0.44, 4.18)	1.73 (0.46, 2.32)	0.836
Outcome			
mTICI (2b‐3), *n* (%)	258 (93.1%)	94 (93.1%)	1.000
Symptomatic HT, *n* (%)	43 (15.5%)	9 (8.9%)	0.128
3‐month mRS (0–1), *n* (%)	86 (31.0%)	41 (40.6%)	0.086
3‐month mRS (0–2), *n* (%)	115 (41.5%)	52 (51.5%)	0.101
Stroke etiology, *n* (%)			
ICAS‐MCAO	123 (44.4%)	42 (41.6%)	0.641
Embolism‐MCAO	154 (55.6%)	59 (58.4%)	

*Note:* *p < 0.05.

Abbreviations: ASPECTS, alberta stroke program early computed tomography score; CT, computed tomography; DBP, diastolic blood pressure; DPT, door to puncture time; DSA, digital subtraction angiography; EIGR, early infarct growth rate; Embolism‐MCAO, middle cerebral occlusion due to embolism; HMCAS, hyperdense middle cerebral artery sign; HT, hemorrhagic transformation; ICAS‐MCAO, middle cerebral artery occlusion due to intracranial atherosclerosis; IQR, Interquartile Range; LMR, lymphocyte to monocyte ratio; MCA, middle cerebral artery; mRS, modified Rankin scale; mTICI, modified Thrombolysis In Cerebral Infarction; NIHSS, National Institute of Health Stroke Scale; NLR, neutrophil to lymphocyte ratio; PLR, platelet to lymphocyte ratio; PRT, puncture to reperfusion time; SBP, systolic blood pressure; SD, standard deviation.

### 4.2 Predictors of Etiology in Patients With MCAO

In the univariate analysis (Table 2), several factors (age, atrial fibrillation, admission SBP, admission DBP, baseline NIHSS scores, HMCAS, ASPECTS classification, onset to CTP time, core volume, mismatch ratio, EIGR, stenosis/occlusion in other arteries, collateral status, and D‐dimer) were found to be associated with etiology in patients with MCAO (*p* < 0.05). In the multivariate logistic regression analysis (Table 3), five variables remained statistically significant for embolism‐MCAO in the training cohort: presence of atrial fibrillation (odds ratio [OR], 46.603 [95% CI, 17.005–127.715]; *p* < 0.001), HMCAS (OR, 3.283 [95% CI, 1.391–7.749]; *p* = 0.007), EIGR ≥ 7.545 (OR, 2.976 [95% CI, 1.283–6.904]; *p* = 0.011), without stenosis/occlusion in other arteries (OR, 4.023 [95% CI, 1.180–13.724]; *p* = 0.026), and poor collateral circulation (OR, 2.941 [95% CI, 1.161–7.447]; *p* = 0.023). These five factors were recognized as independent predictors for etiology before mechanical thrombectomy in MCAO patients and were then used for creating the prediction score.

**TABLE 2:** Comparison of baseline characteristics between atherosclerotic MCAO and embolic MCAO in training cohort.

Variable	ICAS‐MCAO (*n* = 123)	Embolism‐MCAO (*n* = 154)	*p*
Demographic characteristics			
Age (year), median (IQR)	64.0 (52.0, 70.5)	71 (62.0, 80.0)	< 0.001*
Male, *n* (%)	81 (65.9%)	84 (54.5%)	0.065
Medical history			
Hypertension, *n* (%)	87 (70.7%)	91 (59.1%)	0.058
Diabetes, *n* (%)	31 (25.2%)	27 (17.5%)	0.138
History of stroke, *n* (%)			
None	108 (87.8%)	135 (87.7%)	0.318
Ischemic	15 (12.2%)	16 (10.4%)	
Hemorrhagic	0 (0.0%)	3 (1.9%)	
Atrial fibrillation, *n* (%)	9 (7.3%)	125 (81.2%)	< 0.001*
Clinical information			
Admission SBP (mmHg), mean ± SD	133.93 ± 20.31	123.18 ± 19.29	< 0.001*
Admission DBP (mmHg), mean ± SD	77.24 ± 11.71	71.10 ± 14.78	< 0.001*
Baseline NIHSS scores, median (IQR)	13.0 (9.0, 17.5)	17.0 (12.0, 21.0)	< 0.001*
Neuroimaging‐CT			
With HMCAS, *n* (%)	32 (26.0%)	109 (70.8%)	< 0.001*
ASPECTS Classification, *n* (%)			
ASPECTS (0–5 scores)	6 (4.9%)	23 (14.9%)	0.009*
ASPECTS (6–10 scores)	117 (95.1%)	131 (85.1%)	
Onset to CTP time (h), median (IQR)	7.30 (4.16, 12.18)	4.08 (2.23, 6.58)	< 0.001*
Infarct core (mL), median (IQR)	21.97 (4.02, 47.00)	47.00 (24.43, 77.54)	< 0.001*
Penumbra median (mL), (IQR)	87.40 (41.20, 128.92)	98.44 (54.04, 135.00)	0.118
Mismatch ratio, median (IQR)	4.43 (2.86, 16.17)	3.19 (2.04, 4.85)	< 0.001*
EIGR (mL/h), median (IQR)	3.28 (0.39, 9.52)	12.97 (5.30, 26.57)	< 0.001*
Neuroimaging‐DSA			
Occlussion site, *n* (%)			
MCA‐M1	108 (87.8%)	123 (79.9%)	0.203
MCA‐M2	14 (11.4%)	28 (18.2%)	
MCA‐M3	1 (0.8%)	3 (1.9%)	
With stenosis/occlussion in other arteries, *n* (%)	29 (23.6%)	9 (5.8%)	< 0.001*
Collateral circulation evaluation, *n* (%)			
Poor	22 (17.9%)	57 (37.0%)	0.001*
Moderate	50 (40.7%)	56 (36.4%)	
Good	51 (41.5%)	41 (26.6%)	
Admission laboratory data			
NLR, median (IQR)	4.48 (3.01, 7.45)	5.23 (3.38, 8.93)	0.138
LMR, median (IQR)	3.77 (2.78, 4.87)	3.58 (2.22, 4.50)	0.744
PLR, median (IQR)	144.12 (114.16, 199.33)	152.97 (99.26, 216.98)	0.094
Uric acid(mmol/L), median (IQR)	353.80 (290.75, 395.35)	359.94 (303.00, 437.30)	0.162
Fasting glucose (mmol/L), median (IQR)	7.75 (6.43, 8.44)	7.57 (6.33, 8.44)	0.916
Fibrinogen (g/L), median (IQR)	2.70 (2.42, 3.45)	2.70 (2.45, 3.48)	0.851
D‐dimer (mg/L), median (IQR)	0.68 (0.34, 3.44)	1.25 (0.44, 4.19)	0.046*

*Note:* *p < 0.05.

Abbreviations: ASPECTS, alberta stroke program early computed tomography score; CT, computed tomography; DBP, diastolic blood pressure; DSA, digital subtraction angiography; EIGR, early infarct growth rate; Embolism‐MCAO, middle cerebral occlusion due to embolism; HMCAS, hyperdense middle cerebral artery sign; ICAS‐MCAO, middle cerebral artery occlusion due to intracranial atherosclerosis; IQR, Interquartile Range; LMR, lymphocyte to monocyte ratio; MCA, middle cerebral artery; NIHSS, National Institute of Health Stroke Scale; NLR, neutrophil to lymphocyte ratio; PLR, platelet to lymphocyte ratio; SBP, systolic blood pressure; SD, standard deviation.

**TABLE 3:** Logistic regression analysis of risk factors for embolism‐MCAO in training cohort.

Variable	Crude OR (95% CI)	*p*	Adjusted OR (95% CI)	*p*
Age ≥ 70	3.753 (2.239–6.292)	< 0.001*	1.212 (0.470–3.126)	0.691
With atrial fibrillation	54.598 (24.789–120.272)	< 0.001*	46.603 (17.005–127.715)	< 0.001*
Admission SBP > 135 mmHg	3.065 (1.816–5.173)	< 0.001*	2.391 (0.922–6.195)	0.073
Admission DBP > 75 mmHg	2.212 (1.364–3.586)	< 0.001*	1.386 (0.563–3.413)	0.477
Baseline NIHSS scores ≥ 15	3.013 (1.842–4.928)	< 0.001*	1.860 (0.782–4.427)	0.161
With HMCAS	6.888 (4.047–11.724)	< 0.001*	3.283 (1.391–7.749)	0.007*
ASPECTS classification	2.047 (1.105–3.790)	0.023*	1.549 (0.519–4.622)	0.433
0–7 scores				
8–10 scores				
EIGR classification	5.630 (3.351–9.458)	< 0.001*	2.976 (1.283–6.904)	0.011*
< 7.545 (mL/h)				
≥ 7.545 (mL/h)				
Without stenosis/occlussion in other arteries	4.970 (2.252–10.970)	< 0.001*	4.023 (1.180–13.724)	0.026*
Collateral circulation classification	2.698 (1.533–4.748)	0.001*	2.941 (1.161–7.447)	0.023*
Poor				
Moderate‐good				
D‐dimer ≥ 1.095	1.923 (1.185–3.122)	0.008	1.178 (0.505–2.750)	0.704

*Note:* *p < 0.05.

Abbreviations: ASPECTS, alberta stroke program early computed tomography score; CI, confidence interval; DBP, diastolic blood pressure; EIGR, early infarct growth rate; Embolism‐MCAO, middle cerebral occlusion due to embolism; HMCAS, hyperdense middle cerebral artery sign; NIHSS, National Institute of Health Stroke Scale; OR, odds ratio; SBP, systolic blood pressure.

### 4.3 The Risk Score

A risk score (AHEOC score) for embolism‐MCAO, consisting of atrial fibrillation, HMCAS, EIGR, stenosis/occlusion in other arteries and collateral circulation, was developed from the training cohort. Each of the five independent predictors was point assigned based on the β‐coefficient. As a sum of individual points (0–10 points), embolism‐MCAO score includes atrial fibrillation (four points for having atrial fibrillation), HMCAS (two points for having HMCAS), EIGR (1 point for ≥ 7.545 mL/h), stenosis/occlusion in other arteries (two points for having not stenosis/occlusion in other arteries), and collateral status (one point for poor collateral circulation).

Regarding clinical feasibility, EIGR could severely limit the widespread adoption of the AHEOC score. Thereby we developed a simple and universally applicable score which excluded EIGR based on the AHEOC score. Finally, atrial fibrillation (four points for having atrial fibrillation), HMCAS (two points for having HMCAS), stenosis/occlusion in other arteries (one point for having no stenosis/occlusion in other arteries), and collateral status (one point for poor collateral circulation) constructed the AHOC score for predicting embolism‐MCAO (Table 4).

**TABLE 4:** Components of the AHOC score determining middle cerebral occlusion due to embolism or atherosclerosis.

Predictors	AHOC scoring points
With atrial fibrillation	
No	0
Yes	4
With HMCAS	
No	0
Yes	2
Without stenosis/occlussion in other arteries	
No	0
Yes	1
Collateral circulation classification	
Moderate‐good	0
Poor	1

Abbreviation: HMCAS, hyperdense middle cerebral artery sign.

**FIGURE 2:**
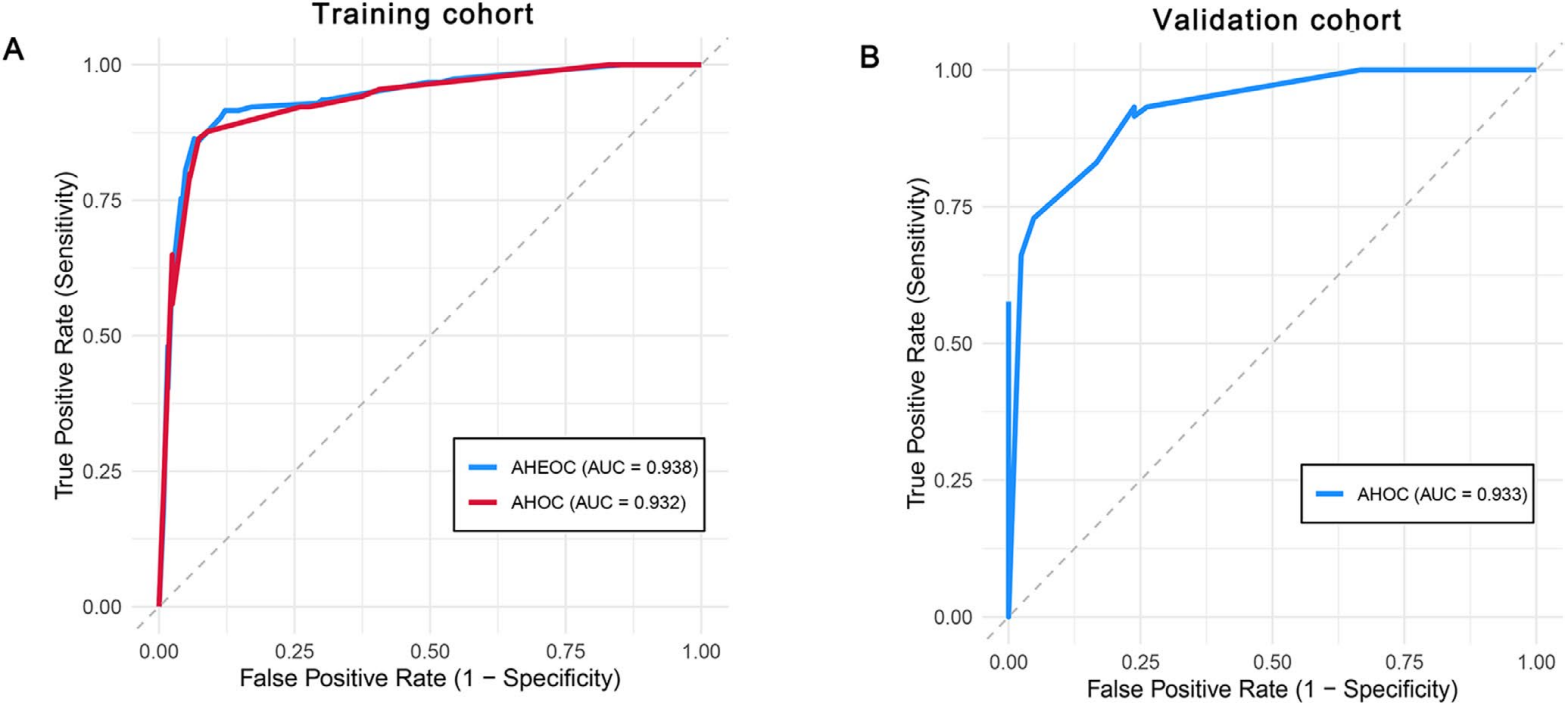
(A) Receiver operating characteristic curves for AHEOC score and AHOC score to predict etiology in the training cohort. (B) Receiver operating characteristic curve for AHOC score to predict etiology in the validation cohort.

Subsequently, we further compared the *C*‐statistics of the two risk scores (AHEOC score and AHOC score), and found no significant difference between the two scores in the training cohort (0.938 [0.908–0.968] vs. 0.932 [0.902–0.963]; *p* > 0.05; Figure 2A). Therefore, we identified AHOC score as the final risk score.

The AHOC score demonstrated good discrimination and calibration in the training cohort (*C*‐statistic, 0.932 [0.902–0.963], Figure 2A; Brier score, 0.092 [0.070–0.115]; *p* value of the Hosmer‐Lemeshow test, 0.604) and in the validation cohort (*C*‐statistic, 0.933 [0.888–0.978], Figure 2B; Brier score, 0.102 [0.067–0.140]; *p* value of the Hosmer‐Lemeshow test, 0.846). The scoring system, using an optimal cut‐off of 3.5 points, demonstrated excellent diagnostic performance. In the training and validation cohorts, the PPV was 92.5% (95% CI: 87.0–95.8) and 95.6% (95% CI: 85.2–98.8), respectively, while the NPV was 85.6% (95% CI: 78.5–90.5) and 71.4% (95% CI: 58.6–81.6), respectively. The sensitivity was 87.7% and 72.9%, and the specificity was 91.1% and 95.2% in the training and validation cohorts, respectively, further confirming the model's strong clinical utility.

**FIGURE 3:**
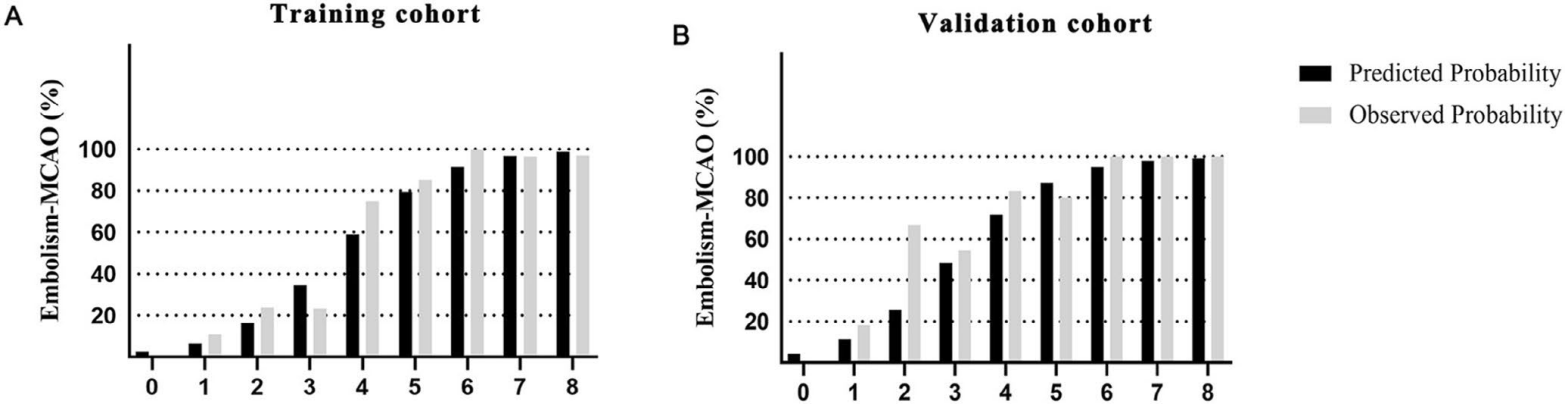
(A) The predicted and observed proportion of patients identified embolism‐MCAO according to the AHOC score in the training cohort. (B) The predicted and observed proportion of patients identified embolism‐MCAO according to the AHOC score in the validation cohort. Embolism‐MCAO, Middle cerebral artery occlusion due to embolism.

The predicted and observed proportion of patients who identified embolism‐MCAO increased with increasing scores in both the training group and the validation group (Figure 3A,B). Based on the AHOC scoring system, high scores were significantly correlated to embolism‐MCAO; alternatively, lower scores were associated with ICAS‐MCAO. 3.5 points was the optimal cutoff value on the predictive scale in the training cohort, demonstrating 87.7% sensitivity, 91.1% specificity, and 78.8% accuracy. Similarly, 3.5 points was the optimal cutoff value on the validation cohort, demonstrating 72.9% sensitivity, 95.2% specificity, and 68.1% accuracy for the diagnosis of embolism‐MCAO.

## 5 Discussion

The MT strategy is obviously influenced by different etiologies. In our study, a novel and simple risk score (AHOC score) composed of atrial fibrillation, HMCAS, stenosis/occlusion in other arteries, and collateral status was established to identify etiology before MT in patients with MCAO. According to the AHOC score, patients with MCAO could be identified as embolism‐etiology or ICAS‐etiology, respectively. For clinical practicality, we focused on parameters that could be conveniently available before intervention. The risk score exhibited good discrimination and calibration in the training and validation cohorts. For a patient stratified into the “High‐risk for embolism‐MCAO category” (a score of 4–8) based on the AHOC scoring system, the predicted etiology is most likely an embolus. In this scenario, a direct aspiration technique as the first‐line approach might be favored. Conversely, for a patient stratified into the “High‐risk for ICAS‐MCAO category” (a score of 0–3), the underlying pathology is an atherosclerotic stenosis with a high risk of re‐occlusion. In this case, a stent retriever‐first strategy might be preferred. Thus, the risk scores could help neurointerventionalists determine the optimal MT strategy before MT.

Some previous studies have developed several prediction models to identify embolic stroke or ICAS stroke in patients with LVO, including CHESS, ICAS‐LVO scale, ATHE scale, and ABC^2^D score [12, 19, 22, 23]. Each of the four predicting models was composed of different predictors, but all of them contained atrial fibrillation, indicating its strong predictive power. In our present study, we also found that atrial fibrillation was the strongest predictor of etiology relative to other markers, and the presence of atrial fibrillation was assigned a score of 4 in the embolism‐MCAO scoring system. On the other hand, a crucial factor is hyperdense artery sign, whose importance is emphasized by its close correlation with embolic LVO [12, 19]. However, the CHESS and ICAS‐LVO scale did not incorporate hyperdense artery sign, probably due to its applicability [22, 23]. It is well‐established that the presence or absence of a hyperdense sign is best evaluated in the MCA, and the hyperdense artery sign has been reported in 17%–50% of patients with MCA territory stroke [16, 24]. However, for other large vessel occlusions, hyperdense sign is not easy to assess, probably causing false negatives. Moreover, the grading of the leptomeningeal collaterals is most appropriate for MCA territory stroke [17, 18]. Most importantly, acute occlusion of the middle cerebral artery is the most common large vessel occlusion stroke [2, 3]. Therefore, we developed and validated a scoring system for etiology before MT in patients with MCAO in our present study. To our knowledge, this is the first model for identifying etiology before MT, purely focusing on MCAO stroke.

Notably, in our initial scoring system (AHEOC score) we included a relatively novel indicator, EIGR, representing early infarct core growth rate. It is well recognized that infarct evolution exhibits remarkable heterogeneity among patients, as some patients may suffer fast growth resulting in large infarcts within a short time, while others may experience slower evolution leading to small infarcts despite a longer time between onset and imaging acquisition [25]. Although the underlying pathophysiology of fast and slow EIGR due to LVO is unclear, patients with robust collateral circulation are more likely to have a slow EIGR [26], whereas patients with a large severe hypoperfusion tend to have a fast EIGR [27]. Sarraj et al. [28] revealed that the EIGR strongly correlated with both collateral status and clinical outcomes in patients with LVO. He et al. [15] suggested the increasing severity of leukoaraiosis was associated with fast EIGR. However, few studies have explored the association of EIGR with the etiology of LVO. In our present study, we found that patients with embolic MCAO had a greater EIGR, which was an independent predictor of embolism‐MCAO. However, the calculation of EIGR relies on CT perfusion imaging and dedicated post‐processing software, which may not be readily available in all primary stroke centers. Additionally, post‐processing software varies, and calculated core and mismatch volumes may vary. Considering clinical applicability and convenience, we attempted to develop a simple scoring system (AHOC score) excluding EIGR, and compared it with the initial AHEOC score and found that the two scores had comparable predictive power for etiology. Therefore, EIGR was not incorporated into our final scoring system (AHOC score).

Our study has several limitations that deserve comment. Firstly, although a retrospective cohort for modeling and a prospective cohort for validation in our study, this was a single‐center study and therefore lacked external validation from other centers. Secondly, we did not compare our scoring system with other predictive models, such as CHESS, ICAS‐LVO scale, ATHE scale, and ABC^2^D score [12, 19, 22, 23]. This decision was influenced by the fact that we purely focused on middle cerebral artery occlusion stroke, and the heterogeneity of the patients among studies may cause no significance for comparison. Thirdly, although our scoring system demonstrated excellent discrimination and calibration in both cohorts, the sample size of the validation cohort (*n* = 101) was relatively modest. This may lead to less precise estimates of model performance, as reflected in the wide confidence interval of the *C*‐statistic (0.888–0.978). Therefore, the results of this external validation should be interpreted as preliminary, and larger, multicenter studies are warranted to confirm the generalizability and robustness of our score.

## 6 Conclusions

Our scoring system encompasses four components: atrial fibrillation, HMCAS, stenosis/occlusion in other arteries, and collateral status and is an accurate and applicable model for predicting the embolic or atherosclerotic etiology in patients with MCAO before MT. This scoring system could assist neurointerventionalists in determining the optimal MT strategy for acute MCAO patients.

## Author Contributions

Concept and design: H.Z., Y.S., Y.F., D.H., J.L.; acquisition of data: Z.X., Y.C., R.Y., W.G., Z.H., L.C., C.Z., Q.H., J.Z., K.Z.; analysis or interpretation of data: H.Z., Y.S., J.H., P.F., X.L.; drafting of the manuscript: H.Z., Y.S.; critical revision of the manuscript for important intellectual content: Y.F., D.H., J.L.; obtained funding: J.L.; All authors have read and approved the final manuscript.

## Funding

This study was supported by the National Natural Science Foundation of China (No. 82460247), the Natural Science Foundation of Jiangxi Province (No. 20212BAB216023), and the Educational Science Foundation of Jiangxi Province (No. GJJ2200136).

## Ethics Statement

This study was approved by the Ethics Committee of the First Affiliated Hospital of Nanchang University (IIT 2023 Clinical Ethic Review No. 364).

## Conflicts of Interest

The authors declare no conflicts of interest.

## Data Availability

Data are available upon reasonable request. All data are available from the corresponding author.
